# TagSNP approach for HLA risk allele genotyping of Saudi celiac disease patients: effectiveness and pitfalls

**DOI:** 10.1042/BSR20210509

**Published:** 2021-06-10

**Authors:** Reham H. Baaqeel, Babajan Banaganapalli, Hadiah Bassam Al Mahdi, Mohammed A. Salama, Bakr H. Alhussaini, Meshari A. Alaifan, Yagoub Bin-Taleb, Noor Ahmad Shaik, Jumana Yousuf Al-Aama, Ramu Elango, Omar I. Saadah

**Affiliations:** 1 Department of Genetic Medicine, Faculty of Medicine, King Abdulaziz University, Jeddah, Saudi Arabia; 2 Princess Al-Jawhara Al-Brahim Center of Excellence in Research of Hereditary Disorders, King Abdulaziz University, Jeddah, Saudi Arabia; 3 Department of Biology, Faculty of Science, King Abdulaziz University, Jeddah, Saudi Arabia; 4 Pediatric Gastroenterology Unit, Department of Pediatrics, Faculty of Medicine, King Abdulaziz University, Jeddah, Saudi Arabia

**Keywords:** autoimmunity, Celiac, Genetics, HLA Typing

## Abstract

Background: Celiac disease (CD) is a genetically complex autoimmune disease which is triggered by dietary gluten. Human leukocyte antigen (HLA) class II genes are known to act as high-risk markers for CD, where >95% of CD patients carry (HLA), *DQ2* and/or *DQ8* alleles. Therefore, the present study was conducted to investigate the distribution of HLA haplotypes among Saudi CD patients and healthy controls by using the tag single nucleotide polymorphisms (SNP).

Methods: HLA-tag SNPs showing strong linkage value (*r*^2^>0.99) were used to predict the HLA *DQ2* and *DQ8* genotypes in 101 Saudi CD patients and in 103 healthy controls by using real-time polymerase chain reaction technique. Genotype calls were further validated by Sanger sequencing method.

Results: A total of 63.7% of CD cases and of 60.2% of controls were predicted to carry HLA-*DQ2* and *DQ8* heterodimers, either in the homozygous or heterozygous states. The prevalence of *DQ8* in our CD patients was predicted to be higher than the patients from other ethnic populations (35.6%). More than 32% of the CD patients were found to be non-carriers of HLA risk haplotypes as predicted by the tag SNPs.

Conclusion: The present study highlights that the Caucasian specific HLA-tag SNPs would be of limited value to accurately predict CD specific HLA haplotypes in Saudi population, when compared with the Caucasian groups. Prediction of risk haplotypes by tag SNPs in ethnic groups is a good alternate approach as long as the tag SNPs were identified from the local population genetic variant databases.

## Introduction

Celiac disease (CD) is an immune-related disorder of gastrointestinal system, which is triggered by ingestion of gluten peptide found in cereals like wheat, rye and barley. Originally, CD was thought to exclusively affect white Europeans [1], but recent reports indicate its increasing prevalence in diverse ethnic groups like Caucasians, Africans, Arabs and South Asians [2–6]. This increasing frequency of CD could be attributed to the rapid changes in lifestyle and diet and also due to the recent developments in diagnostic procedures. Recent studies indicates that the prevalence of CD ranges from 0.6% to 1.1% among the Middle Eastern arab countries [7]. Although CD is considered a major health problem in the Middle Eastern region, exact frequency of CD remains elusive due to the lack of large scale data [2]. Patients with classical CD presents gastrointestinal (GI) manifestations like diarrhea, malabsorption, abdominal pain and distension, bloating, vomiting, and weight loss [3,8–10]. Currently, gluten-free food is the standard dietary restriction to manage the disease complications [11].

The strongest genetic predisposing factors known to explain 25–40% of CD’s heritability are, human leukocyte antigen DQ (HLA-DQ) class II haplotypes, which are formed by variants in the highly polymorphic HLA -*DQA1* and -*DQB1* genes [12,13]. HLA genes encode cell surface receptors of most antigen presenting cells, which forms a cleft that binds to gliadin peptides. The genetics of the various HLA haplotypes that contribute to CD development is complex, as the disease risk is basically determined by the number and configuration of the *DQA1* and *DQB1* alleles. About 90–95% of CD patients share HLA-*DQ2* heterodimer (encoded by HLA-DQA1*0501 and HLA-DQB1*02 alleles) and the remaining patients carry HLA-*DQ8* heterodimer (encoded by HLA-DQA1*0301 and HLA-DQB1*0302 alleles) [14]. It is extremely rare for individuals negative for both *DQ2* and *DQ8* risk alleles to develop CD [15]. Therefore, due to its very high negative predictive value, HLA typing has become a standard exclusion criteria in CD diagnosis [6,15,16]. Even though HLA-DQ haplotypes are major predisposing genetic factors, they are not sufficient to develop the disease because only 20–30% of the normal population carry these HLA-DQ variants [17,18]. This fact supports the contribution of other HLA and non-HLA genetic loci regions in CD predisposition [17,19,20].

Traditionally, CD linked HLA risk variants are genotyped by PCR-based HLA typing using sequence-specific oligonucleotide probes (SSOP), sequence-specific primers (SSP), and Sanger sequencing-based typing (SBT) methods. No doubt that these methods have improved the HLA typing, but several inherent limitations like time-consuming and expensive protocols, low throughput, unphased data and ambiguity of results limits their widespread use in molecular diagnosis. In this regard, Monsuur et al. has developed a simple, high-throughput allelic discrimination method to rapidly predict the *DQ2.5, DQ2.2, DQ7*, and *DQ8* risk alleles using tagSNPs. This tag SNP approach has shown >94.0% predictive value for CD diagnosis, with >96.8% of sensitivity and >99.4% of specificity, when tested among European population [14]. This method was then eventually used for population screening to determine the prevalence of CD HLA risk alleles in few other ethnic groups [14,21–25].

To the best of our knowledge, data on the distribution of HLA locus gene variants and their relevance to CD diagnosis among Saudi population are limited. Therefore, our study is aimed to assess the utility of real-time PCR based tagSNPs to provide new information on HLA-DQ risk haplotypes associated with CD in Saudi Arabia. Moreover, the present study has also aimed to investigate the distribution of these HLA risk alleles among CD patients and healthy population in Saudi Arabia.

## Materials and methods

### Recruitment of study subjects and sampling

Ethical approval for this study was granted from the Research Ethics Committee, King Abdulaziz University Hospital (KAUH), Jeddah. Unrelated Saudi nationals with CD were recruited from a Pediatric Gastroenterology clinic; all cases were examined at the Department of Genetic Medicine for obtaining information about the prevalence of disease among other family members and the comorbidities. A total of 101 sporadic CD patients were clinically diagnosed based on the guidelines of European Society for Paediatric Gastroenterology, Hepatology and Nutrition (ESPHGAN) which includes serology testing for antibodies against gliadin and endomysium (EMA) or tissue transglutaminase (tTG) followed by small bowel biopsy to confirm the diagnosis for serology positive results [26].

Additionally, 103 healthy Saudi controls, who are over 20 years old, having no personal or family history for allergies, autoimmune or inflammatory disorders (Diabetes, Rhematic arthritis, and Systemic Lupus Erythematosus) were randomly recruited from volunteers. All the enrolled participants and the parents of the children (<16 years) were informed about the study processes before obtaining written informed consent to participate in the present study. We collected 5 ml of peripheral blood samples in EDTA vacutainers from all participants and stored at −20°C until DNA extraction procedure was conducted.

### Genotyping

#### Genomic DNA isolation

We isolated DNA from the 200 μl blood samples with QIAamp DNA Mini Kit (Catalogue # 51306). DNA Quality and quantity measurements were done using NanoDrop™ 2000c Spectrophotometer.

#### Genotyping for HLA-DQ tag SNPs

**Figure 1:**
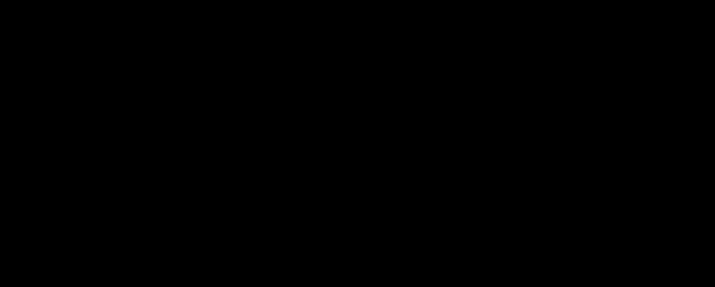
Tag SNP positions with reference to HLA loci (in kb) (DQ2.2) rs2395182 + rs7775228: *r*^2^ = 0.971, (DQ2.5) rs2187668: *r*^2^ = 0.994, (DQ8) rs7454108: *r*^2^ = 0.892.

TaqMan®Genotyping assay (Applied Biosystems) was run by using 7500 FAST Real-Time PCR machine (Applied Biosystem, Int., U.S.A.). to genotype the individuals for the four HLA tag SNPs (rs7775228, rs2395182, rs2187668, and rs7454108) listed in Table 1 with chromosomal positions shown in Figure 1. TaqMan®Genotyper Software (Applied Biosystem, int. U.S.A.) was used to analyze scatter plots of test samples.

**Table 1:** Selected HLA TagSNPs associated with Celiac disease

Detected HLA Haplotype	SNP ID	Gene	Variant Type	Chr. Position	Allele Change	TaqMan Assay ID
HLA-DQ2.2	rs2395182	HLA-DRA	intergenic variant	6:32445540	[G/T]	C__29315313_10
	rs7775228	HLA-DQB1	regulatory region variant	6:32690302	[T/C]	C__11409965_10
HLA-DQ8	rs7454108	NR	regulatory region variant	6:32713706	[T/C]	C__58662585_10
HLA-DQ2.5	rs2187668	HLA-DQA1	intronic	6:32638107	[C/T]	C__29817179_10

NR, not reported.

#### HLA genotype notation using the tagSNPs

The genotype status of the tagSNPs is used to interpret HLA-DQ haplotypes as presented in Table 2. As per the original study published the Mansuur et al. [14], the homozygous (for major or minor allele) or heterozygous (both major and minor alleles) status of the queried tag SNP will predict the individual’s HLA-DQ haplotype status due to its strong Linkage Disequilibrium (LD) value seen among Caucasian population (*r*^2^ value >0.99).

**Table 2:** Interpretation of HLA tagSNPs to HLA-DQ alleles genotypes

SNP	Genotype	DQ2.5 Type	SNP	Genotype	DQ8 Type
**rs2187668**	CC	DQx	rs7454108	CC	DQ8/DQ8
**rs2187668**	CT	DQ2.5/DQx	rs7454108	CT	DQ8/DQx
**rs2187668**	TT	DQ2.5/DQ2.5	rs7454108	TT	DQx
-					
**SNP**	**Genotype**	**rs7775228/ DQ2.2 Type**	**rs7775228/ DQ2.2 Type**	**rs7775228/ DQ2.2 Type**	
		**TT**	**CT**	**CC**	
**rs2395182**	GG	DQx	DQx	DQx	
**rs2395182**	GT	DQx	DQ2.2/DQx	DQ2.2/DQx	
**rs2395182**	TT	DQx	DQ2.2/DQx	DQ2.2/DQ2.2	

* DQX, not DQ2.2, DQ2.5, DQ8.

The determination of HLA-DQ2.5 and DQ8 haplotypes is straight forward and is predicted by the genotype status of rs2187668 and rs7454108 SNPs, respectively. Whereas *DQ2.2* haplotype was determined based on the genotype status of 2 tagSNPs i.e., ‘T’ (major allele) for rs2395182 and ‘C’ (minor allele) for rs7775228. The homozygous statuses of TT (rs2395182) and CC (rs7775228) for both the tag SNPs suggest that the individual is homozygous to *DQ2.2* haplotype. In case of *DQ2.2* heterozygous haplotype, individuals will have either of the GT-CT, GT-CC or TT-CT genotype combinations for the rs2395182 and rs7775228 tag SNPs. The individuals who are not carrying either ‘T’ (rs2395182) or ‘C’ (rs7775228) alleles in the abovementioned combination were considered to be negative for *DQ2.5/DQ2.2* and *DQ8* haplotypes and carrying a different HLA-DQ haplotype (DQx), Table 2.

### Validation of genotype calls

To check the accuracy and reproducibility of SNP genotyping assay, we performed Sanger sequencing of 100 random DNA samples from both CD patients and controls and compared the results between these two approaches. Prior to Sanger sequencing, the PCR products were purified using QIA quick PCR Purification Kit following the manufacturer instructions (Qiagen, Alameda, CA, U.S.A.). Purified PCR products were used as a DNA template for cycle sequencing reactions using ABI 3500 Genetic Analyzer (Life Technologies, U.S.A.). The reaction mixture of cycle sequencing PCR consists of 1 μl of big dye, 2 μl of 5× big dye buffer, 1 µl of either forward or reverse primer, and 1 μl of purified PCR product and 5μl nuclease-free water. Bioedit software 6 version was used for alignment and identifying the sequence variants.

### Statistical analysis

To assess the CD risk conferred by different HLA genotypes, we conducted the analysis using Statistical Package for Social Sciences (SPSS) software version 14.0. Statistically significant difference in allele and genotypes was determined using Pearson’s standard chi-squared test, odds ratio (OR), and 95% confidence interval (CI) and *P* value <0.05 was considered significant.

## Results

### Clinical analysis

The present study included a total of 101 CD patients (45 males and 56 females), and the 103 healthy controls (43 males and 60 females). The mean age of patients was found to be 28.8 ± 13.9 years and for controls it was 31.6 ± 8.8 years. Of the study participants, 30% of the patients and 50% of the controls were born to consanguineous parents. The common clinical symptoms seen in CD patients were as follows: chronic diarrhea, abdominal pain, anorexia and abdominal distension. The common autoimmune manifestations seen in our patient group were type 1 diabetes mellitus (32%), autoimmune thyroiditis (8%), and systemic lupus erythematosus (3%). We also observed few non-autoimmune disease manifestations like osteomalacia (5%), seizure disorders (4%), and Down syndrome (4%).

### Real time PCR genotyping results

#### HLA tagSNPs- allelic frequency distribution analysis

In Table 3, of the 4 HLA-tag SNPs tested, only three (rs2395182, rs7775228, and rs2187668) have shown the significant difference in minor allele frequency distribution among CD patients in comparison with healthy controls. Our statistical results for rs2395182 have indicated that the minor ‘G’ allele is more prevalent in healthy controls (18%) than in CD group (10%) [*P*=0.015, OR = 2.01, 95% CI = 1.138–3.562]. For rs7775228 SNP, significant differences in the frequency of minor ‘C’ allele among controls (21%) and CD patients (14%) was observed [*P*=0.0497; OR = 1.67; 95% CI = 0.998–2.787]. Interestingly, for the rs2187668, 10% of CD group were found to be carrying the minor ‘T’ allele compared with the 3% of the healthy controls. This difference in minor allelic frequency is statistically significant [*P*=0.001; OR = 4.074; 95% CI = 1.615–10.273]. For rs7454108, there was no statistically significant difference between case and control groups (*P*-value > 0.5).

**Table 3:** Comparison of HLA-DQ tagging SNPs allele frequencies

rs ID	Alleles		Frequency		OR	95%CI	X2	*P*-value
			Cases (*n*=101)	Control (*n*=103)				
rs2395182	Minor	G	0.104	0.189	2.013	[1.138–3.562]	5.925	0.015^*^
	Major	T	0.896	0.811				
rs7775228	Minor	C	0.144	0.218	1.667	[0.998–2.787]	3.852	0.048^*^
	Major	T	0.856	0.782				
rs7454108	Minor	C	0.188	0.18	0.945	[0.572–1.56]	0.049	0.824
	Major	T	0.812	0.82				
rs2187668	Minor	T	0.1	0.03	4.074	[1.615–10.273]	10.157	0.001^*^
	Major	C	0.9	0.97				

* Values are statistically significant *P* ≤0.05.

#### HLA-DQ haplotype results

The risk classification of the HLA-DQ genotypes (Table 4) is based on previous study from Saudi Arabia [27]. In our study, tag SNP predicted homozygous HLA-*DQ2.5* haplotype is significantly high in CD patients compared to healthy controls (10.89 vs 2.91%; p = 0.024). No heterozygous *DQ2.5* (in combination with *DQ8* or *DQ2.2*) carriers were predicted by the tag SNP combinations in either patient or control groups. The *DQ8* heterozygous haplotypes (*DQ8/DQ2.2* and *DQ8/DQX*) were highly frequent among CD patients (33.66%) with significant difference in *DQ8/DQX* haplotype between patients and control group, (32.67% vs 14.56% respectively, p = 0.014). Surprisingly, the homozygous *DQ8* high risk haplotype was predicted by the tag SNPs more frequently in control groups (7.76%) than in patients (1.98%). However, the difference was not statistically significant. The predicted homozygous HLA-*DQ2.2* haplotype was also frequent in control (5.82%) group than in CD patients (0.99%). A total of 29.13% controls and 24.75% CD patients were heterozygous for *DQ2.2*. A total of 74 (36.27%) individuals predicted to be lacking all the high risk HLA alleles and seen as the extremely low risk group (*DQX*).

**Table 4:** HLA-DQ Genotyping Frequency by TagSNP Method

	Haplotype	Controls (*n*) = 103	Cases (*n*) = 101	OR	CI	*P-*Value
Very high risk	DQ2.5/DQ8	–	–	–	–	NA
High risk	DQ2.5/DQ2.5	(3) 2.91	(11) 10.89	4.07	(1.1015–15.0691)	0.024^*^
	DQ2.5/DQ2.2	–	–	–	–	NA
	DQ8/DQ8	(8) 7.76	(2) 1.98	0.24	(0.0497–1.1587)	0.556
Intermediate risk	DQ2.5/DQX	–	–	-	–	NA
	DQ8/DQ2.2	(6) 5.83	(5) 4.95	0.84	(0.2486–2.8518)	0.782
Low Risk	DQ8/DQX	(15) 14.56	(29) 32.67	2.36	(1.1771–4.7434)	0.014^*^
	DQ2.2/DQ2.2	(6) 5.82	(1) 0.99	0.16	(0.0191–1.3678)	0.578
	DQ2.2/DQX	(24) 23.3	(20) 19.8	0.81	(0.4161–1.5875)	0.543
Very low risk	DQX	(41) 39.81	(33) 35.67	73.00%	(0.4137–1.3018)	0.289

NA: Not Applicable ∗Values are statistically significant *P*≤0.05.

### Validation by sanger sequencing results

**Figure 2:**
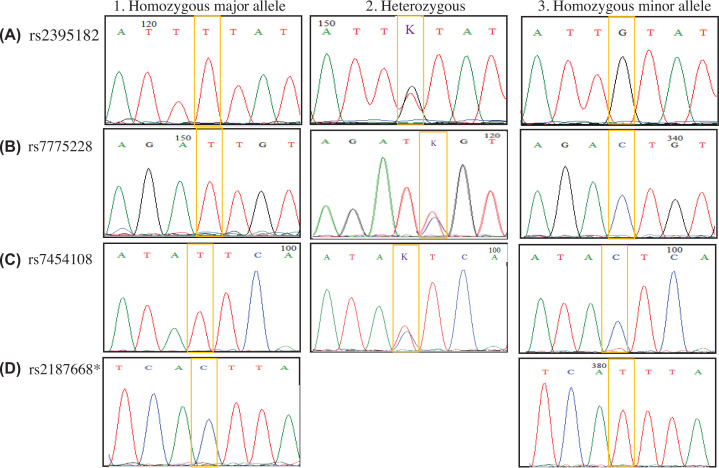
Sanger sequencing results for the 4 HLA tagSNPs used in the present study * No heterozygous form was detected for SNP (rs2187668).

The sequencing analysis with sanger method showed the accuracy (100%) of Real-time PCR based tagSNPs approach in determining the HLA-DQ haplotype among randomly selected individuals for each SNP genotypes from both study groups (Figure 2).

## Discussion

In the present study, we assessed the transferability of the Real-time PCR based TaqMan SNP Genotyping Assay to accurately predict the HLA risk haplotypes associated with CD using four of the six tag SNPs in the Saudi population for the first time. Two HLA-tagSNPs (rs4713586 for *DQ4* and rs4639334 for *DQ7*) were withdrawn from the present study because they were not polymorphic in the Saudi population (as per data from Saudi Human Genome Project -SHGP) and are not useful in tagging the targeted HLA haplotypes.

Our results shows that, in total 67.3% of Saudi CD patients were predicted to be the carriers of HLA CD-associated major risk alleles. We observed that Saudi individuals with homozygous HLA-*DQ2.5* haplotype have a 4-fold higher risk to develop CD (OR = 4.074).This finding further confirms previous studies, which revealed a high risk associated with two copies of *DQ2.5* among Europeans, Africans and Arabs (Table 5) [17,22,24,27–31]. A recent study among Saudi children has reported that the homozygous *DQ2.5* was seen in more CD cases than healthy controls. In that study, presence of either HLA-*DQ8* or HLA-*DQ2.2* alone did not confer a risk of CD in the Saudi children; however, the combination of *DQ2.5* with either *DQ8* or *DQ2.2* significantly increases the disease risk in general population [27]. In contrast, no heterozygous *DQ2.5* individuals have been predicted in this study. It can be explained by the low minor allele frequency for the predictive ‘T’ allele of the tagging rs2187668 SNP in this population (0.1%, 0.03%) in CD patients and control respectively, (Table 3).

**Table 5:** HLA Risk haplotype for Celiac disease distribution among Middle Eastern countries

		Risk haplotype frequency (%)												
		Highest	High			Intermediate		Low			Very Low	^*^Overall frequency (%)		Reference
**Population**	**Groups**	**DQ2.5/DQ8**	**DQ2.5/DQ2.5**	**DQ2.5/DQ2.2**	**DQ8/DQ8**	**DQ2.5/DQX**	**DQ8/DQ2.2**	**DQ8/DQX**	**DQ2.2/DQ2.2**	**DQ2.2/DQX**	**DQX/DQX**	**DQ2**	**DQ8**	
Egypt	Cases *n*=31	16.13	41.94	6.45	9.68	12.9	9.68	NR	NR	3.23	3.25	77.42	35.49	Mohammed, M., et al. 2014 [31]
Iran	Cases *n*=59	11.9	13.6	11.9	3.3	27.1	0	8.5	1.7	5.08	3.3	64.5	23.7	Rostami-Nejad, M., et al. 2014 [22]
	Control *n*=151	3.3	0.6	3.3	2.6	6.6	5.3	7	0	14.5	21.2	13.8	18.2	
Israel	Cases *n*=44	9	NR	NR	NR	NR	NR	NR	NR	NR	4.5	66	20.5	Pallav, K., et al.,2014 [43]
	Control *n*=173	4.6	NR	NR	NR	NR	NR	NR	NR	NR	57.8	61.8	22.5	
Jordan	Cases *n*=44	NR	NR	NR	NR	NR	NR	NR	NR	NR	NR	80	NR	El-Akawi, Z., 2015 [44]
	Control *n*=53	NR	NR	NR	NR	NR	NR	NR	NR	NR	NR	32	NR	
Libya	Cases *n*=31	NR	NR	32	3	52	10	NR	NR	3	NR	84	13	Alarida, K., et al. 2010 [37]
	Control *n*=156	3.2	NR	7.7	12.2	36	4.5	NR	6.4	19.2	14.7	59.6	19.9	
Morocco	Cases *n*=115	4.3	19.1	26.1	0	12.2	1.7	7	1.7	6.1	0	61.7	13	Piancatelli, D., et al. 2017 [29]
	Control *n*=96	7.3	3.1	3.1	0	18.8	2.1	14.6	1	12.5	22.9	32.3	24	
Gaza strip Palestine	Cases *n*=65	4.6	NR	NR	NR	NR	NR	NR	NR	NR	7.9	70.8	15.4	Ayesh, et al. 2017 [40]
	Control *n*=97	3.1	NR	NR	NR	NR	NR	NR	NR	NR	NR	17.5	27.8	
Saudi Arabia	Cases *n*=101	0	10.89	0	1.98	0	3.96	29.7	0.99	16.83	35.6	10.89	35.64	Present study
	Control *n*=103	0	2.91	0	7.76	0	3.88	15.5	5.82	17.47	46.6	2.91	27.14	
Saudi Arabia	Cases *n*=100	11	12	17	4	39	6	8	1	2	0	79	29	Al‐Hussaini, A., et al. 2019 [27]
	Controls *n*=192	0	2.6	4.7	4.2	28.15	3.6	9.4	9.4	3.6	20.8	35.45	17.2	
Syria	Cases *n*=49	10.2	49	10.2	0	0	2	8.2	4.1	0	0	69.4	20.4	Murad, H., et al. 2018 [24]
	Control *n*=58	1.7	10.3	3.4	0	0	1.7	3.4	5.2	0	60	15.4	6.8	
Tunisia	Cases *n*=94	NR	NR	NR	NR	NR	NR	NR	NR	NR	NR	84	NR	Bouguerra, F., et al.,1997 [45]
Turkey	Cases *n*=78	28.2	35.8	NR	11.5	15.3	NR	6.4	NR	NR	2.6	78.5	46.1	Çakır, M., et al. 2014 [46]
	Control *n*=13	15.3	38.4	NR	15.3	7.6	NR	15.3	NR	NR	NR	61.3	45.9	

NR, Not Reported/specific allele statues is not clear in the published articles.

* Overall frequency for (a) DQ2 encoded by DQA1*0501 and DQB1*02 alleles either in homozygous or heterozygous state. (b) DQ8 encoded by DQA1*0301 and DQB1*0302 alleles either in homozygous or heterozygous state.

Based on our HLA predictions and from previous studies, it is clear that the frequency of the HLA haplotypes in Arabs is not necessarily different from Europeans, but tagSNPs used for Caucasian population may not accurately predict the HLA genotype in this population. Significant differences in the patterns of linkage disequilibrium in the genomes of Saudis with the highest consanguineous marriages in the world might explain limited benefit of using the same tagSNPs to predict the heterozygote HLA*-DQ2.5* allele. Several studies have shown that social factors such as the differences in consanguinity levels in populations is responsible for changes in genotype frequencies and results in the loss of heterozygosity with increasing homozygous genotypes [32–34]. First cousin marriages among Arabs is high and the overall rates of consanguinity in Saudi Arabia ranges from 52.1% to 67.7% for many generations in the past [34,35]. The frequency of consanguinity within our study is 30% in CD patients and 50% in controls.

We found that the most frequent predicted haplotype among Saudi CD patients was *DQ8* (33.66%) in heterozygous form with a statistically significant *P*=0.014 for *DQ8/DQX* that increases the possible risk by more than 2 folds than the general population (OR = 2.36). Similar high frequency of HLA-*DQ8* is seen in CD patients and Amerindian groups in Chile [36]. Our findings showed a significant difference of predicted *DQ8* among CD patients in comparison with other Arab population listed in Table 5, as well as other populations such as Cameroon, Italy, Hungary, United States, Finland and Japan [23,37] with low frequency of heterozygo*tes*. Furthermore, among healthy Saudi children fewer heterozygote *DQ8/DQX* and homozygous *DQ8* haplotypes were reported [27]. This finding suggests that the tag SNP rs7454108 in our study predicting excessively more *DQ8* haplotype in the Saudi population than the other studies which used classical HLA genotyping methods.

Previous study reported about 17–20% of the general Saudi population carry HLA-*DQ8*, higher than in the Caucasians, 1–9% [35,38]. These differences propose that variable combinations of HLA-DQ risk alleles among Saudi CD patients might confer different risk gradients for some HLA-DQ molecules compared to Caucasian CD patients. Although homozygous *DQ8* haplotype is considered to be strongly CD-associated high risk molecule [39], surprisingly in our population, it was seen more frequently predicted in healthy controls (7.76%) than in CD patients (1.98%). Such differences, though not significant, were seen in Finnish, Hungarian and in some Arab population like Gaza strip [23,40].

The *DQ2.2* haplotype is a low risk haplotype in CD patients in many countries [21,24,41,42]. In our study, predicted homozygous *DQ2.2* was more frequent in control group (5.82%) than in cases (0.99%). Although *DQ2.2* is known to raise the risk for CD when associated with *DQ2.5* or *DQ8*, it did not confer the high risk for CD in our population. This low frequency of predicted *DQ2.2* among CD patients may suggest it plays a minor role in triggering the autoimmune process in our population [42]. This finding was also supported by Al-Hussaini's study that *DQ2.2* alone did not confer the risk for CD in the Saudi children [27]. This might also suggest the protective role of *DQ2.2* allele in the Saudi population, which requires to be tested in a larger study. High frequency of DQA1*02:01 allele that is associated with HLA-*DQ2.2* haplotype in Santiago, Chile among control subjects suggests that they protect the population against the CD development [36]. However, it was not possible in our study to determine whether predicted *DQ2.2* individuals were carrying the DQA1*02:01 allele or not because of the low frequency of the predictive minor allele of tagSNPs in this population.

To date, most HLA data on CD patients studies has come from only few Arab countries, that too on a smaller sample size. In Table 5 our predicted HLA genotype frequency among CD patients and across different Arab countries shows some similarities as well as differences.

The present study has provided information on the predicted HLA genetic background of CD in Saudi population. The differences in HLA’s association with CD as observed in the present study population compared to non-Arab populations could be due to the different ethnic and cultural practices like first cousin marriages, which will in turn influence the polymorphic nature of SNPs. The present study also suggests that transferability of tagSNP approach in populations (like Arabs, African, Japanese and Chinese etc.) which have known differences in LD structure, still needs to be determined. Therefore, an immediate search for other tag SNPs with higher *r*^2^ value for disease association needs to be identified using population specific genetic database such as SHGP.

In conclusion, tagSNP typing is a reliable and easy alternate approach to rapidly genotype highly polymorphic HLA region. The present study represents the first investigation to test the applicability of tag SNPs to determine the HLA status of CD patients in Saudi population. Our findings reveal that, tag SNPs predicted homozygous *DQ2.5* and heterozygous *DQ8* haplotypes, of HLA are associated with CD development among Saudi patients. The findings of this study highlight that Caucasian specific tagSNPs would be of limited value to accurately predict CD specific HLA haplotypes in Saudi population. More than 32% of the CD patients were predicted to be not carrying the HLA risk alleles, highlighting the low predictive value for them in Saudi population. Large-scale HLA typing of Arab CD patients with different highly polymorphic population specific tagSNPs might reveal the accurate picture of HLA risk haplotypes in the disease diagnosis and treatment.

## Data Availability

The data generated by us are presented in the form of tables and figures in the manuscript. Individual participant SNP genotypes or HLA haplotypes cannot be released due to the Institutional ethical committee rules and regulation to protect the privacy of the participants and to maintain the confidentiality of their clinical information. All the pooled data is presented in the manuscript to protect the privacy of the participants and maintain the confidentiality of their personal data.

## Abbreviations

χ2: Chi-square
bp: Base Pair
CD: Celiac Disease
CI: Confidence Interval
DGP: De-amidated Gliadin Peptide Antibody
DNA: Dexoyribonucleic Acid
dNTP: Deoxyribonucleotide Triphosphate
EDTA: Ethylenediaminetetraacetic Acid
EMA: Anti-endomysium
ESPHGAN: European Society for Paediatric Gastroenterology, Hepatology and Nutrition
GFD: Gluten Free Diet
GI: Gastrointestinal
GWAS: Genome-Wide Association Studies
HLA: Human Leukocyte Antigen
IgA/IgG: Anti-Gliadin Antibodies
KAUH: King Abdulaziz University Hospital
LD: Linkage Disequilibrium
MHC: Major Histocompatibility Complex
OR: Odds Ratio
PCR: Polymerase Chain Reaction
SHGP: Saudi Human Genome Program
SNP: Single-Nucleotide Polymorphism
SPSS: Statistical Package for the Social Sciences
tTG: Anti-Transglutaminase
